# Identification of linear human B-cell epitopes of tick-borne encephalitis virus

**DOI:** 10.1186/1743-422X-11-115

**Published:** 2014-06-19

**Authors:** Suvi Kuivanen, Jussi Hepojoki, Sirkka Vene, Antti Vaheri, Olli Vapalahti

**Affiliations:** 1 Department of Virology, Haartman Institute, Faculty of Medicine, University of Helsinki, Helsinki, Finland; 2 Department of Virology and Immunology, Helsinki University Central Hospital Laboratory (HUSLAB), Helsinki, Finland; 3 Department of Veterinary Biosciences, Faculty of Veterinary Medicine, University of Helsinki, Helsinki, Finland; 4 The Public Health Agency of Sweden, Solna, Sweden

**Keywords:** Epitope, Flavivirus, Tick-borne encephalitis virus

## Abstract

**Background:**

Tick-borne encephalitis (TBE) is a central nervous system infection transmitted to humans by ticks. The causative agent, tick-borne encephalitis virus (TBEV), belongs to the genus Flavivirus (family *Flaviviridae)*, which includes globally important arthropod-borne viruses, such as dengue, Yellow fever, Japanese encephalitis and West Nile viruses. Flaviviruses are highly cross-reactive in serological tests that are currently based on viral envelope proteins. The envelope (E) protein is the major antigenic determinant and it is known to induce neutralizing antibody responses.

**Methods:**

We synthesized the full-length TBEV proteome as overlapping synthetic 18-mer peptides to find dominant linear IgG epitopes. To distinguish natural TBEV infections from responses to TBE immunization or other flavivirus infections, the peptides were probed with sera of patients infected with TBEV, West Nile virus (WNV) or dengue virus (DENV), sera from TBE vaccinees and negative control sera by SPOT array technique.

**Results:**

We identified novel linear TBEV IgG epitopes in the E protein and in the nonstructural protein 5 (NS5).

**Conclusions:**

In this study, we screened TBEV structural and nonstructural proteins to find linear epitopes specific for TBEV. We found 11 such epitopes and characterized specifically two of them to be potential for differential diagnostics. This is the first report of identifying dominant linear human B-cell epitopes of the whole TBEV genome. The identified peptide epitopes have potential as antigens for diagnosing TBEV and to serologically distinguish flavivirus infections from each other.

## Introduction

Tick-borne encephalitis virus (TBEV) is one of the most important neurotropic arthropod-borne pathogens in Europe, causing nearly 3000 hospitalizations annually [1]. TBEV is endemic in many European countries, Russia and China. It is mainly transmitted by *Ixodes* ticks, but can also be transmitted via consumption of unpasteurized goat milk [2,3]. There are three subtypes of TBEV circulating in geographically distinct areas; namely European, Siberian, and Far Eastern. The variation at amino acid level is up to 5.6% between subtypes and 2.2% within a subtype [4].

The flavivirus virion is approximately 50 nm in diameter and displays icosahedral symmetry. The virion consists of three structural proteins (capsid C, prM and envelope E), the RNA genome, and a lipid membrane derived from the host cell. The flavivirus genome is a single-stranded positive-sense RNA (approximately 11 kilobases), which is encapsidated by the C protein. The genome contains a single open reading frame, which encodes a polyprotein that is co- and post-translationally cleaved into ten proteins by viral and host proteases. The envelope protein E is a class II viral fusion protein. It consists of three distinct domains (I, II and III), and forms homodimers in a head to tail manner. In the virion, the homodimers further arrange into trimers parallel to each other. The other envelope protein, prM, is cleaved by furin during viral maturation and the pr moiety is released as a result of conformational changes. The seven nonstructural proteins (NS1, NS2A, NS2B, NS3, NS4A, NS4B and NS5) are found in the infected cell. NS1 is the only nonstructural protein that is glycosylated and secreted outside the cell. NS3 and NS2B form the viral serine protease that is required for post-translational modification of the polyprotein [5]. NS5 is a multifunctional protein containing an N-terminal methyl transferase domain and a C-terminal RNA-dependent RNA polymerase domain [6,7]. The other small nonstructural proteins (NS2A, NS4A and NS4B) are expected to function at least in the genome replication [8].

To date, the E and NS1 proteins are known to raise protective antibodies in infected humans, monkeys and mice [9]. PrM, does not elicit protective antibodies, but is probably required for the preservation of conformational epitopes of the E protein [10]. Previous studies suggest that infections with dengue (DENV), Japanese encephalitis (JEV) and West Nile (WNV) viruses can be differentiated by the antibody response to the prM protein [10,11]. For DENV type 1, some of the dominant epitopes in E and NS1 proteins have been identified using protein fragmentation methods [9]. In addition, AnandaRao *et al.* characterized several immunodominat linear B-cell epitopes in C and NS4A proteins of DENV using multi-pin peptide synthesis strategy [12].

In the present study, we used a peptide-based approach to identify immunodominat linear B-cell epitopes from the entire TBEV genome, which have not been previously reported. We found TBEV-specific peptides in the E and NS5 proteins. The characterized epitopes showed potential in differentiating between other flavivirus infections, and between natural and vaccine-derived immunity to TBEV.

## Results

### Proteome-wide epitope screening

**Figure 1:**
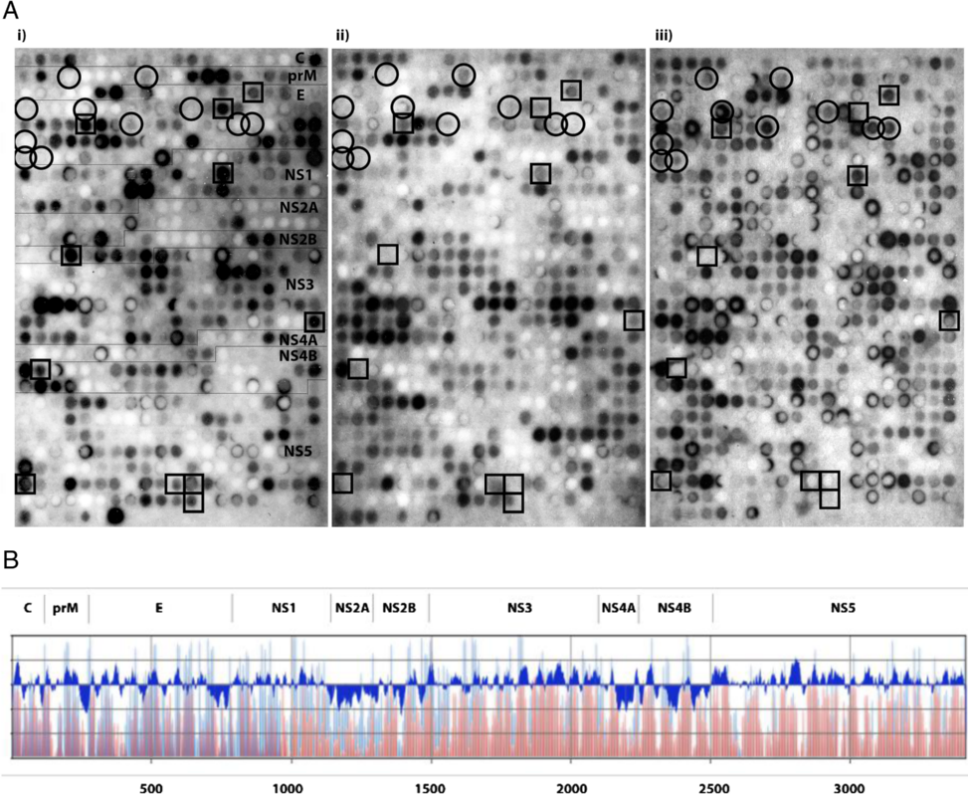
**SPOT array of TBEV proteome as overlapping peptides and prediction of antigenic regions. A)** SPOT array of TBEV coding region, acute TBEV-seropositive pool spots in rectangle, TBEV-immunized spots in circles, i) acute TBEV-seropositive serum pool, ii) TBEV-seronegative serum pool and iii) TBEV-immunized serum pool. **B)** Kyte and Doolittle hydrophilicity plot of the TBEV genome corresponds to the signal intensities from the TBEV-seropositive and –negative serum pools. The window in Kyte and Doolittle hydrophilicity plot is 18. Amino acids in X-axis, signal intensities of the SPOT array in Y (TBEV-seropositive in blue, TBEV-seronegative in red), hydrophilicity (above) and hydrophobicity (below) in the upper X-axis.

A total of 567 overlapping linear 18-mer peptides were initially probed with pools of TBEV-seronegative, acute TBEV-seropositive sera, and a pool of sera from TBEV-immunized individuals. Several epitope regions were identified in both structural and nonstructural parts of the TBEV proteome as highlighted by squares (peptides positive with either TBEV seropositive or TBEV-immunized pool) and circles (peptides positive only by TBEV-immunized pool) in Figure 1A. We used densitometry to quantify the signal intensities of the peptides given by each pool, and plotted the signal intensity on a graph in parallel with Kyte and Doolittle hydrophilicity plots (Figure 1B). This quite expectedly showed that the antigenic regions overlapped with the hydrophilic regions throughout the proteome. To study which of the epitopes are dominant in TBEV-seropositive individuals, we probed the membrane next with individual serum samples of the TBEV seropositive serum pool. Peptides that were positive in at least 4/5 of the acute-phase samples, but remained negative in densitometric quantification with seronegative pool, were selected for further analysis (highlighted by squares in Figure 1A). In total, we identified 11 such IgG epitopes throughout the TBEV proteome (Table 1).

**Table 1:** Proteome-wide TBEV-specific linear epitope mapping

**Protein**	**Index**	**Virus**	**Peptide sequence**	**Sensitivity**	**Vaccinated**
**E**	56	TBEV	E N P A K T R E Y C L H A K L S D T	4/5	**+**
**E**	74	TBEV	G R K T A S F T V S S E K T I L T M	4/5	**-**
**E**	85	TBEV	L P W K H E G A Q N W N N A E R L V	5/5	**+**
**NS1**	154	TBEV	E C P L E R R K T G V F T V A E F G	4/5	**-**
**NS2B**	244	TBEV	A G L A A S A I H W S G I L G V M G	4/5	**+**
**NS3**	340	TBEV	P W L A W H V A A N V S S V T D R S	4/5	**-**
**NS4B**	382	TBEV	S E W T N V D I Q P A R S W G T Y V	4/5	**-**
**NS5**	521	TBEV	A L N T L T N I K V Q L I R M M E G	4/5	**+**
**NS5**	531	TBEV	P L D D R F G K A L Y F L N D M A K	4/5	**-**
**NS5**	532	TBEV	G K A L Y F L N D M A K T R K D I G	4/5	**-**
**NS5**	552	TBEV	W S I H A S G A W M T T E D M L D V	5/5	**-**

### Epitope characterization

To further fine-map the identified eleven peptide epitopes, a new set of overlapping 18-mer peptides with a single amino acid shift were synthesized from a region including six additional amino acids in both N- and C-termini of the parent peptide, resulting in a total of 13 peptides per each originally selected peptide. To evaluate the potential cross-reactivity between flaviviruses, we included the region corresponding to the parent peptide from heterologous flaviviruses (Table 2). The novel array was probed individually with a new panel of paired sera from six TBE-patients and 20 TBEV-seronegative sera (Table 3). With these samples, three of the original 11 peptides remained positive, two in E (E-74/38 and E-85/59) and one in NS5 (NS5-521/198), when using a limit of at least 4/6 TBE-patients positive (in either acute or convalescent sample) and no more than 2/20 of TBEV-seronegative samples positive (Table 4). Additionally, we probed the peptides with DENV-seropositive (serotypes 1–4) and WNV-seropositive (four pools, four sera in each) sera. None of the three positive peptides reacted with DENV or WNV seropositive sera, which reacted with some of the DENV and WNV peptides, respectively.

**Table 2:** Virus strains used for heterologous peptide synthesis

**Virus**	**Strain**	**GeneBank accession number**
TBEV	European	GU183380
DENV1	Singapore S275/90	M87512
DENV2	New Guinea C	AF038403
DENV3	strain H87	M93130
DENV4	-	AY94753
LGTV	-	NC_003690
OHFV	-	NC_005062
LIV	-	NC_001809
WNV	Rabensburg isolate 97-103	AY765264
WNV	isolate WN NY 2000-crow3356	AF404756
JEV	-	NC_001437
USUV	-	NC_006551
MVEV	-	NC_000943
YFV	17D vaccine	NC_002031

**Table 3:** Paired TBEV-seropositive serum samples

**Sample**	**Sample ID**	**IgG**	**IgM**	**NT**	**E-74/38**	**E-85/59**	**NS2B- 244/124**	**NS5- 521/197**	**NS5- 521/198**	**NS5- 521/206**
Acute	GG15-44	+	+	<5	**-**	**+**	+	**-**	**-**	+
Convalescent	GG15-339	+	-	40	**+**	**+**	+	**+**	**+**	+
Acute	GG5-36	+	+	<5	**+**	**+**	-	**-**	**+**	-
Convalescent	GG5-198	+	-	40	**-**	**+**	-	**+**	**+**	-
Acute	GG12-62	+	+	5	**+**	**+**	+	**+**	**+**	+
Convalescent	GG12-286	+	-	160	**+**	**+**	+	**-**	**+**	+
Acute	GG38-126	+	+	<5	**+**	**+**	+	**+**	**+**	+
Convalescent	GG38-128	+	+	5	**-**	**+**	+	**-**	**-**	-
Acute	GG33-102	+	+	<5	**-**	**-**	-	**-**	**-**	-
Convalescent	GG33-344	+	-	20	**-**	**-**	+	**-**	**-**	-
Acute	GG57-194	+	+	<5	**-**	**+**	-	**-**	**+**	+
Convalescent	GG57-590	+	-	40	**-**	**+**	+	**+**	**+**	+

NT: neutralization titer. TBEV peptides in bold.

**Table 4:** Specificity of the selected peptide epitopes

**Protein**	**Index**	**Virus**	**Peptide sequence**	**TBEV**	**Neg. controls**	**Vaccinated**	**WNV pools**	**DENV (1, 2, 3 or 4)**	**Overall sensitivity**
E	56/7	TBEV	E N P A K T R E Y C L H A K L S D T	3/6	2/20	3/8	2/4	2/4	7/11
E	74/38	TBEV	A S F T V S S E K T I L T M G E Y G	4/6	2/20	1/8	0/4	0/4	8/11
E	85/59	TBEV	L A L P W K H E G A Q N W N N A E R	5/6	1/20	0/8	0/4	0/4	10/11
NS1	154/88	TBEV	E C P L E R R K T G V F T V A E F G	3/6	13/20	2/8	1/4	0/4	7/11
NS2B	244/124	DENV2	G L L V I S G L F P V S I P I T A A	5/6	2/20	0/8	2/4	1/4	9/11
NS3	340/142	TBEV	P W L A W H V A A N V S S V T D R S	6/6	9/20	2/8	2/4	1/4	10/11
NS4B	382/169	TBEV	S E W T N V D I Q P A R S W G T Y V	1/6	9/20	0/8	2/4	0/4	5/11
NS5	521/197	TBEV	L N T L T N I K V Q L I R M M E G E	5/6	2/20	3/8	0/4	0/4	9/11
NS5	521/198	TBEV	N T L T N I K V Q L I R M M E G E G	5/6	2/20	1/8	0/4	0/4	9/11
NS5	521/206	DENV3	G L N T F T N M E A Q L I R Q M E G	4/6	2/20	7/8	0/4	0/4	8/11
NS5	531/223	TBEV	P L D D R F G K A L Y F L N D M A K	4/6	6/20	1/8	0/4	0/4	8/11
NS5	532/250	TBEV	G K A L Y F L N D M A K T R K D I G	2/6	10/20	1/8	0/4	0/4	6/11
NS5	552/277	TBEV	W S I H A S G A W M T T E D M L D V	3/6	8/20	1/8	0/4	0/4	8/11

**Figure 2:**
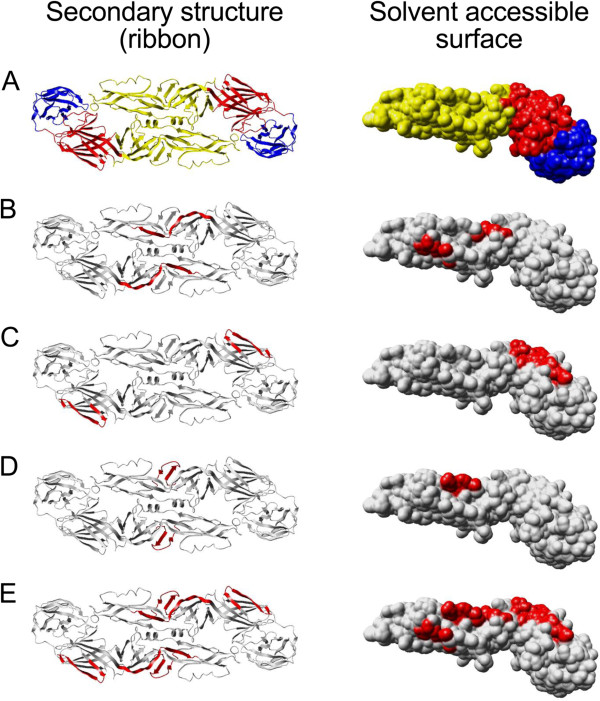
**Location of the identified peptide epitopes in E protein structure.** Left-hand panels show the secondary structure (dimer) and right-hand panels show the solvent accessible surface (monomer) of E protein. **A)** TBEV E protein dimer as viewed from above (the virion) colored (domain I in red, domain II in yellow, and domain III in blue) according to Rey F *et al*. (Rey et al. [13]). **B)** Peptide E-56/7 (residues 51–58, in domain 2), highlighted in red. **C)** Peptide E-74/38 (residues 163–180, in domain 1), highlighted in red. **D)** Peptide E-85/59 (residues 223–240, in domain 2), highlighted in red. **E)** Peptides E-56/7, E-74/38, and E-85/59, highlighted in red.

**Figure 3:**
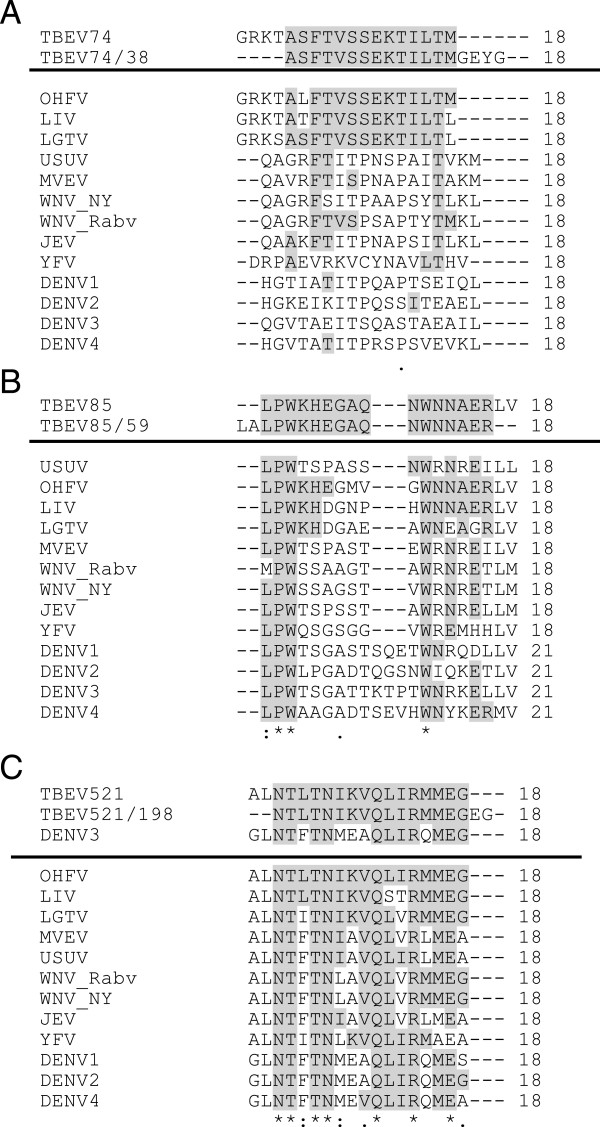
**Clustal 2.1 multiple sequence alignment of the synthesized heterologous peptides with A) TBEV E-74 B) TBEV E-85 C) TBEV NS5-521.** The line separates peptides reactive with TBEV-seropositive sera (above) from the non-reactive ones. Identical amino acids in grey. Conserved amino acids marked with asterisks.

Of these peptides, the peptide E-74/38 (aa 443-460/aa 162–179 of E) lies in the domain I and the peptide E-85/59 (aa 503-520/aa 222–239 of E) in the domain II of the E protein (Figure 2). Amino acid residues in peptide E-85/59 fell into a region that has been identified in low pH-induced fusion with endosomal membrane (residues 221–240) [13,14]. Based on peptide sequence alignments, the minimal amino acid sequence for the peptide E-74/38 is 14 amino acids and it differed from the other tick-borne flaviviruses by a minimum of one C-terminal residue (Figure 3A). However, as shown in Figure 2C, peptide E-74/38 represents a region with two antiparallel beta-sheets, and thus most likely forms a conformational epitope. For the peptide E-85/59, the minimal sequence recognized is 16 amino acids and it shows very little or no homology to other flavivirus heterologous peptides (Figure 3B). Also, the reactivity towards the peptide E-85/59 represents most likely a response towards a conformational epitope formed by a protruding loop of domain II in TBEV E protein (Figure 2D). Peptide E-56/7 was found to cross-react with DENV and WNV sera pools. This peptide is located in domain II of the E protein (Figure 2A) and represents a long stretch of two beta sheets. This peptide would be only partially accessible in the virion, and most likely the C- and N-terminal portions of this peptide (separately) are involved in formation of a conformational epitope with other regions in domain II (Figure 2A).

**Figure 4:**
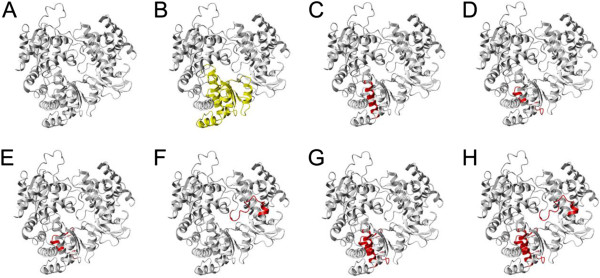
**Three-dimensional model of TBEV NS5 structure with the identified peptide epitopes. A)** Ribbon diagram of TBEV NS5 model. **B)** Palm domain of TBEV NS5 model (according to analogy to WNV NS5 (Malet *et al*., [28]), highlighted in yellow. **C)** Peptide NS5-521/198 (residues 612–629), highlighted in red. **D)** Peptide NS5-531/223 (residues 670–687), highlighted in red. **E)** Peptide NS5-532/250 (residues 676–683), highlighted in red. **F)** Peptide NS5-552/277 (residues 796–813), highlighted in red. **G)** Peptides NS5-521/198, NS5-531/223, and NS5-532/250, highlighted in red. **H)** Peptides NS5-521/198, NS5-531/223, NS5-532/250, and NS5-552/277, highlighted in red.

The third identified linear epitope NS5-521/198 was in the C-terminal RNA-polymerase region of the NS5 protein (aa 3123-3140/aa 612–629) [6]. The minimal amino acid sequence for NS5-521/198 (Figure 3C) differs by a minimum of two amino acids from other tick-borne flaviviruses. The parent peptide 521 is identical to the corresponding peptide from Omsk hemorrhagic fever virus (OHFV, Figure 3C). However, the parent peptide yielded only borderline positive values in quantitation whereas more clearly positive values with less cross-reactivity were obtained with peptide NS5-521/198 containing two additional amino acids. Since a 3D structure is available for the NS5 of several flaviviruses, excluding TBEV, we used homology modeling to generate a model for TBEV NS5 structure. The peptide NS5-521/198 (Figure 4C) is located in the palm domain of TBEV NS5 (Figure 4B, residues 499–541 and 610–717). In addition, two of the other peptide epitopes (NS5-531/223 and NS5-532/250, Figure 4D and E, respectively, excluded due to cross-reactivity) in NS5 lie in the palm domain. The third dominant, but rather cross-reactive, epitope in NS5 (peptide NS5-552/277) is located in the thumb domain in the TBEV NS5 structure model (Figure 4F).

### Linear epitopes in natural versus vaccine-induced TBEV immunity

Finally, to compare the B-cell epitope responses induced by vaccination and infection, we probed the new membrane with eight serum samples of persons immunized and showing neutralizing antibody responses against TBEV. None of the eight sera recognized the peptide E-85/59, suggesting that fixation used in virus inactivation might alter the conformation of this potentially conformational epitope (Figure 2D).

One out of eight sera recognized the peptide E-74/38 that also likely represents a conformational epitope (Figure 2C, Table 4). The results summarizing the new membrane using individual serum samples are given in Table 4. Curiously, quite a strong cross-reactivity with a peptide from DENV3 NS5 (corresponding to the peptide NS5-521/206) was seen with the serum of several TBEV-immunized individuals (Table 4). Only 1/8 TBEV-immunized serum samples recognized the NS5-521/198 peptide (Table 4). Many of the sera from TBEV-immunized individuals reacted with peptide epitopes that were not recognized by sera of TBEV-infected and TBEV-seronegative individuals (Table 5). These peptides, mostly located in the E protein, could be rendered more antigenic due to fixation required for virus inactivation.

**Table 5:** Peptides specific for TBEV-immunized individuals

**Protein**	**Index**	**Peptide sequence**
prM	24	Q V R V E N G T C V I L A T D M G S
prM	29	I D Q G E E P V D V D C F C R N V D
E	61	T L A E E H Q G G T V C K R D Q S D
E	65	C G L F G K G S I V A C V K A A C E
E	72	T G D Y V A A N E T H S G R K T A S
E	88	E F G A P H A V K M D V Y N L G D Q
E	95	S G H V T C E V G L E K L K M K G L
E	96	E V G L E K L K M K G L T Y T M C D
E	101	S G H D T V V M E V T F S G T K P C
E	121	L G G A F N S I F G G V G F L P K L
E	122	S I F G G V G F L P K L L L G V A L

## Discussion

Currently, TBE diagnostics is based on assays measuring IgM and IgG antibody responses to purified viral particles or consisting of structural proteins, or in some cases recombinant virus-like particles [15,16]. TBEV E-protein is highly cross-reactive among other members of the genus Flavivirus and thus the identification of TBEV-specific antigenic regions would be beneficial. The cross-reactivity between flaviviruses is mainly due to conformational epitopes, and therefore linear epitopes presented as peptides could represent a useful alternative for traditionally used purified virus or recombinant protein diagnostic antigens. Ideally, such antigens could enable differential diagnostics between flaviviruses, and perhaps also between natural TBEV infection and vaccine responses. On the other hand, the potential of the nonstructural proteins as antigens is currently not fully explored. Our study provides one holistic approach for screening antigenic areas with diagnostic potential. For mosquito-borne flaviviruses, Wong *et al.* have developed an NS5-based serological test for differentiating natural WNV-infection from other flavivirus infections (specifically Saint Louis encephalitis and dengue virus) [17].

In this study, we describe the mapping of linear B-cell IgG epitopes in the TBEV proteome. Initial mapping using TBEV-seronegative and acute TBEV-seropositive serum pools revealed several regions containing linear epitopes throughout the TBEV proteome. Further analysis of the epitope regions using individual serum samples revealed that most of the epitopes were not recognized by all of the positive samples. After data analysis, we selected 11 peptides in total from E, NS1, NS2B, NS3, NS4B and NS5 proteins for more thorough characterization. In addition, we addressed the cross-reactivity of TBEV seropositive sera against the corresponding regions from other flaviviruses. Eventually, we identified three TBEV-specific epitope regions that reacted with the majority of TBEV seropositive sera. Two of the identified peptides are in the E protein and one in the NS5 protein region. Of these, peptide E-85/59 showed the highest specificity and potential in serological differentiation between natural and vaccine-derived immunity. Holzmann *et al*. used a similar type of approach to study linear epitopes in TBEV E protein utilizing mouse monoclonal antibodies. Their results demonstrated that only few of the MAbs reacted with synthetic peptides from TBEV E protein. Curiously, these MAbs bound only TBEV E protein only if directly coated on microtitre plate or otherwise denatured [18]. However, in our study we were able to demonstrate several linear epitopes throughout TBEV E protein. Peptide E-85/59, the region identified by Holzmann *et al*. as an epitope for TBEV MAb, was recognized only by sera of infected individuals and not by sera of vaccinated individuals.The space-filling model of the E protein (Figure 2) demonstrates that the identified peptides lie on the surface of the folded protein. Furthermore, both E-74/38 and E-85/59 that are located in domains I and II, respectively, actually form or are parts of a conformational epitope (Figure 2C and D). Neither of these peptides was reactive with sera of TBEV-immunized individuals, thus suggesting that the conformational epitopes at these regions are affected by the fixation used for virus inactivation. Furthermore, we observed that the sera of TBEV-immunized individuals recognized several epitopes in the E protein that were less reactive with sera of naturally infected TBEV-seropositive individuals. This probably highlights the fact that the natural B-cell response towards TBEV, and perhaps towards flaviviruses in general, is based on conformational epitopes. Although vaccination using formalin-fixed inactivated virions is effective, stronger responses would obviously be obtained with replication in cells or with native proteins.

We generated a homology model for TBEV NS5 to visualize the epitopes found in this region. The peptides NS5-521/198, NS5-531/223 and NS5-532/250 identified in this study, are located in the palm domain in the TBEV NS5 structure model. Of these peptides, NS5-521/198 is identical to the respective region in Omsk hemorrhagic virus NS5. The parent peptide reacted (positive with 6/6 of TBE patient and 3/20 of seronegative samples) rather similarly with the OMSKV peptide (positive with 4/6 of TBE patient and 3/20 of seronegative samples). Thus it seems evident that OMSKV-seropositive serum would also recognize this peptide epitope. However, since a couple of epitopes are located in the palm domain of TBEV NS5, one could envision using this region as recombinant antigen for flavivirus diagnostics.

Throughout our study we used densitometry to quantify the signal intensities of the individual peptides on the SPOT array. However, since the detection of SPOT binding is based on an enzymatic reaction, many factors contribute to the signal intensity (incubation and exposure times, membrane regeneration, antibody dilutions, ECL reagents, etc.). Quantification can therefore be done only inside the array and not between individual serum samples. Due to this, we evaluated each membrane individually, and considered peptides positive if the intensity was above a certain assay-specific threshold value. However, this method brings out a challenge in selecting the true-positive peptides, and vice versa, it is likely to miss some potential spots under the arbitrary threshold. As exemplified by a slight discrepancy in the results obtained with DENV2 (3/6 were positive) and DENV3 (4/6 were positive) peptides having identical sequences. This discrepancy is directly explained by the selected threshold values and the factors described above. Additionally, the regeneration steps required for re-use of the SPOT peptide array membranes are not ideal for high throughput screening. Thus we will, in the near future, synthesize some of the promising peptides found in this study in soluble form, and use them as antigens in an enzyme-linked immunosorbent assay (ELISA) format. ELISA will allow us to further test these peptides with larger serum panels and at different dilutions.

## Conclusions

In conclusion, we have for the first time characterized the linear human B-cell epitopes of TBEV using TBE-patient and cross-reacting flavivirus sera. The TBE patients’ sera detect peptide targets particularly on the surface of E and NS5 proteins, and two of these peptides seem promising in distinguishing between TBEV and other flavivirus infections or TBEV vaccine derived immunity.

## Methods

### Computer analysis

The putative hydrophobic and hydrophilic areas of the coding region of TBEV European subtype strain Kumlinge A52 genome (GenBank accession number GU183380) [19,20] were predicted by the Kyte and Doolittle method using MacVector software.

### Peptide synthesis

The whole coding region of TBEV genome strain Kumlinge A52 was synthesized onto an amino-PEG membrane in 567 18-amino acid peptides with six amino acid transition [21]. Based on the initial screening with 567 peptides covering the proteome of TBEV, we selected 11 peptides (Table 1) for further evaluation. Six amino acids were added to both N- and C-terminus of these peptides, which were then synthesized as 18-amino acid peptides with one amino acid transition. Heterologous peptides corresponding to the “parent” peptide (Table 2) were synthesized from other tick- and mosquito-borne flaviviruses. Peptide synthesis was conducted with a MultiPep platform (Intavis Ag) utilizing Fmoc chemistry as described earlier [22].

### SPOT method

The membrane containing the complete proteome of TBEV was initially probed with a pool of sera from TBEV seronegative patients (N = 5), next with a pool of sera from TBEV-immunized individuals (N = 8), then with a pool of sera from TBE patients (N = 5), and finally with individual serum samples collected from acute-phase TBE patients (N = 5). The membrane with peptides selected based on initial screening (11 “parent” peptides, Table 1) was probed individually with six paired serum samples from acute-phase TBE patients and with sera from 8 TBEV-immunized individuals. The probing and detection of SPOT membranes was done as previously described [23]. Briefly, the sera were used at 1:500 dilution (in pools the total serum dilution was 1:500). The binding of antibodies was detected by HRP-labeled anti-human IgG (DakoCytomation) at 1:1000 dilution and the results were visualized utilizing enhanced chemiluminescence as recorded on X-ray film (Fuji RX medical).

### Serum samples

Five acute-phase TBEV-seropositive and five TBEV-seronegative patient sera were selected from serum samples sent to and tested at the Helsinki University Central Hospital Laboratory (HUSLAB), Department of Virology and Immunology. TBEV-seropositive sera were IgM and IgG positive in routinely used serological tests.

The panel of 6 paired serum samples of acute TBE patients and eight serum samples of TBEV-immunized people were collected in Sweden. These sera were characterized by neutralization inhibition at The Public Health Agency of Sweden, Solna, Sweden. The samples of immunized individuals were taken before and after the third vaccine dose of the TBEV vaccination programme. Both sample sets were found TBEV IgM and IgG positive in routinely used serological tests at HUSLAB, Department of Virology and Immunology.

Four pools of WNV patient sera were obtained through ENIVD network (European Network on Improted Viral Diseases, http://www.enivd.de). A total of four DENV IgM and IgG positive patient serum samples (each representing primary infections with a different serotype, DENV-1-4), as determined by RT-PCR [24] were selected from patient samples sent to and tested at HUSLAB, Department of Virology and Immunology.

### Densitometric analysis of peptide intesinty

The results of SPOT analysis recorded on X-ray film were scanned using flatbed scanner (CanoScan LIDE 70) as TIFF files. The SPOT intensities were quantitated using Chemidoc XRS (Bio-Rad). Specific threshold values were assigned for each array individually.

### Protein structures and homology modelling

To visualize the peptide epitopes discovered, we generated a three-dimensional model for TBEV A52 Kumlinge NS5 protein structure using Swiss-Model in Automatic Modelling Mode [25]. Swiss-Model chose WNV NS5 protein (pdb code 2HFZ) [26-29] with a sequence identity of 62.1% to TBEV NS5 as the template for model building. The generated model had a QMEAN6 score of 0.652 with a Z-Score of −1.227 [30]. Based on the QMEAN6 score, we estimated the model to be accurate enough for visualization of the peptide epitopes.

The TBEV E protein structure (1SVB) was obtained from the protein data bank. The domains of E protein were colored as previously described [13] using YASARA [31]. The epitope peptides were highlighted in both TBEV NS5 model and in TBEV E protein structure using YASARA.

## Competing interests

The authors declare that they have no competing interests.

## Authors’ contributions

SK participated in the study design, carried out the SPOT arrays, analyzed the data and drafted the manuscript. JH conceived of the study and participated in its design, synthesized the peptides, contributed in the interpretation of the data, and helped to draft the manuscript. SV characterized the panels of serum samples and revised the manuscript. AV participated in the study design and revised the manuscript. OV participated in the study design, contributed in the interpretation of the data and helped to draft the manuscript. All authors read and approved of the final manuscript.
